# Assessment of the Barriers and Enablers of the Use of mHealth Systems in Sub-Saharan Africa According to the Perceptions of Patients, Physicians, and Health Care Executives in Ethiopia: Qualitative Study

**DOI:** 10.2196/50337

**Published:** 2024-03-27

**Authors:** Genet Tadese Aboye, Gizeaddis Lamesgin Simegn, Jean-Marie Aerts

**Affiliations:** 1 M3-BIORES (Measure, Model & Manage Bioreponses) Division of Animal and Human Health Engineering, Department of Biosystems KU Leuven Leuven Belgium; 2 School of Biomedical Engineering Jimma Institute of Technology Jimma University Jimma Ethiopia

**Keywords:** barriers, chronic disease, digital health, eHealth, enablers, health care, mHealth, mobile health, perspectives, Sub-Saharan Africa

## Abstract

**Background:**

Digital technologies are increasingly being used to deliver health care services and promote public health. Mobile wireless technologies or mobile health (mHealth) technologies are particularly relevant owing to their ease of use, broad reach, and wide acceptance. Unlike developed countries, Sub-Saharan Africa experiences more challenges and obstacles when it comes to deploying, using, and expanding mHealth systems. In addition to barriers, there are enabling factors that could be exploited for the design, implementation, and scaling up of mHealth systems. Sub-Saharan Africa may require tailored solutions that address the specific challenges facing the region.

**Objective:**

The overall aim of this study was to identify the barriers and enablers for using mHealth systems in Sub-Saharan Africa from the perspectives of patients, physicians, and health care executives.

**Methods:**

Multi-level and multi-actor in-depth semistructured interviews were employed to qualitatively explore the barriers and enablers of the use of mHealth systems. Data were collected from patients, physicians, and health care executives. The interviews were audio recorded, transcribed verbatim, translated, and coded. Thematic analysis methodology was adopted, and NVivo software was used for the data analysis.

**Results:**

Through this rigorous study, a total of 137 determinants were identified. Of these determinants, 68 were identified as barriers and 69 were identified as enablers. Perceived barriers in patients included lack of awareness about mHealth systems and language barriers. Perceived enablers in patients included need for automated tools for health monitoring and an increasing literacy level of the society. According to physicians, barriers included lack of available digital health systems in the local context and concern about patients’ mHealth capabilities, while enablers included the perceived usefulness in reducing workload and improving health care service quality, as well as the availability of mobile devices and the internet. As perceived by health care executives, barriers included competing priorities alongside digitalization in the health sector and lack of interoperability and complete digitalization of implemented digital health systems, while enablers included the perceived usefulness of digitalization for the survival of the highly overloaded health care system and the abundance of educated manpower specializing in technology.

**Conclusions:**

mHealth systems in Sub-Saharan Africa are hindered and facilitated by various factors. Common barriers and enablers were identified by patients, physicians, and health care executives. To promote uptake, all relevant stakeholders must actively mitigate the barriers. This study identified a promising outlook for mHealth in Sub-Saharan Africa, despite the present barriers. Opportunities exist for successful integration into health care systems, and a user-centered design is crucial for maximum uptake.

## Introduction

eHealth, or the secure and cost-effective application of information and communication technology (ICT) to support health and health-related sectors, includes, but is not limited to, the use of mobile wireless technologies for public health or mobile health (mHealth) [1,2]. Digital technologies are increasingly being used to deliver health care services and promote public health. mHealth technologies are particularly relevant due to their ease of use, broad reach, and wide acceptance [3]. Global health service delivery could change as a result of the use of mHealth to support the accomplishment of health objectives. This transformation is being fueled by a potent confluence of different elements. These include the quick development of mobile technologies and apps, the rise of new chances for incorporating mHealth into already existing eHealth services, and the ongoing expansion of mobile cellular network coverage [4,5].

In the literature, it has been shown that mHealth systems in the form of SMS text messages, apps, and telemedicine projects are being used efficiently in the developed world. mHealth solutions are advancing rapidly in these regions [6-8]. The reason for this is the fact that there are a number of enabling factors that facilitate or encourage the use and implementation of such systems. Today, a wide variety of barriers and enablers are present in the developing world that influence the use and implementation of mHealth systems [9-11].

The prevalence and exploitation of mHealth technologies are steadily rising, presenting a significant opportunity for their incorporation into clinical services as a means to enhance the provision of high-quality medical care. Recent data on mobile phone usage in Sub-Saharan Africa revealed that 51% of residents possess a mobile device [12]. The latest statistics on mobile service subscriptions suggest an ongoing upward trajectory, with expectations of further increases. As of the end of 2020, 495 million individuals, equivalent to 46% of the population in Sub-Saharan Africa, held active mobile service subscriptions. Projections anticipate that this figure will rise, with an estimated 50% of the population (equivalent to 619 million individuals) anticipated to subscribe to mobile services by the end of 2025. Presently, there are 303 million internet users in Sub-Saharan Africa, constituting 28% of the population, and this number is projected to increase to 474 million by 2025 [12,13]. These data underscore the widespread penetration of mobile technology in the region and lay a solid foundation for exploring the potential advantages and challenges associated with its integration into health care services.

Even though the mass penetration of mobile phones is a fact and a very substantial condition to adopt mHealth systems in such countries, there are a number of factors that need to be considered when designing and developing mHealth systems in developing countries. Most mHealth systems initiated in such countries remain in the proof of concept or pilot testing stage. The large implementation of such systems is not widely documented [14,15].

mHealth approaches are widely embraced, and the health care system heavily relies on them in developed regions. From patient management systems to individual patient-centered mobile apps, these initiatives have been operational for a considerable period, in contrast to their counterparts in developing nations. A minor portion of Sub-Saharan African countries, less than half of the total [16,17], are currently endeavoring to incorporate mHealth platforms into their health care systems. This underscores the requirement for additional development of mHealth technology in the region. Ethiopia is currently focusing on strengthening its health care system and aligning it with the Sustainable Development Goals [18]. With a largely rural population, the nation encounters difficulties in accessing essential services like health care among others. A potential path for improvement lies in the digitalization of health care services, offering the opportunity to enhance efficiency, accessibility, and overall health care outcomes [19].

The implementation of digital health systems may encounter various challenges, and these challenges are generally context-specific. There is no universal solution that can address these challenges in all circumstances. Given the variation in challenges and the absence of a one-size-fits-all solution, success factors center on designing an mHealth platform that is specific to the context and the target population. This involves identifying these determining factors and incorporating sensitive design considerations to address unique needs [13]. Various studies have been performed for understanding technology adaption factors using various frameworks such as the Technology Acceptance Model (TAM) and the Unified Theory of Acceptance and Use of Technology (UTAUT) [20-22]. The research conducted by Liu et al [21] using the UTAUT indicated that users’ intentions to adopt mHealth systems are positively influenced by factors such as effort expectancy, performance expectancy, subjective norm, and perceived ubiquitousness. Notably, privacy concerns exhibited a significantly negative impact only on perceived ubiquity, with no significant effects observed on effort expectancy, performance expectancy, subjective norm, and intention to adopt [21]. The study by Yang et al [20] examined consumers’ intentions and behaviors related to the use of digital applications based on the UTAUT and provided valuable guidance for broadening the use of mHealth apps among consumers.

A systematic research study by Jacob et al [23] comprehending the sociotechnical factors influencing patients’ acceptance of mHealth tools proposed adopting a patient-centric strategy by ensuring that the tools seamlessly integrate into the overall patient journey and treatment plan. This involves giving priority to inclusive design and ensuring thorough patient education and support. Different frameworks have been used to evaluate the implementation of mHealth approaches, but they fall short of addressing all aspects comprehensively. In response, researchers have put forth a consolidated framework to address this limitation by incorporating various factors such as organizational and policy factors, social and personal factors, and technical and material factors [24].

The digital divide, characterized by unequal access to digital technology, is another factor that may be worsened or improved through the adoption of mHealth [25-27]. Despite evidence showing increased access to mobile devices, there is insufficient implementation of mHealth in Sub-Saharan Africa. Accelerating the integration of digitalization into the heavily burdened health care system could help address the challenges associated with inadequate health care and contribute to narrowing the digital divide in the region. Sub-Saharan Africa presents a unique challenge when it comes to the implementation and scaling up of mHealth systems. In contrast to developed countries, the region faces significant barriers that hinder the adoption and effective use of these systems. However, despite the numerous obstacles facing mHealth adoption in Sub-Saharan Africa, there are also opportunities and enabling factors. Consequently, the design and implementation of mHealth interventions in Sub-Saharan Africa must be tailored to address these specific challenges. Ethiopia, a representative country of Sub-Saharan Africa, is considered as one of these nations with limited implementation and consumption of mHealth systems. Thus, there is a pressing need for a comprehensive study of the barriers and enablers for mHealth adoption in Sub-Saharan Africa in order to help guide the development of tailored mHealth interventions that are suited to the local context and can effectively address the unique challenges facing the region. For this, it is vital to investigate the determinants that could affect the use of mHealth approaches. mHealth strategies are tailored to diverse end users, with certain approaches adapted for organizational use, while others target health care professionals or patients, and sometimes both. The factors influencing each of these populations may exhibit overlaps, yet they are varied and complex, necessitating a comprehensive and separate investigation for each category.

The overall aim of this study was to identify the barriers and enablers from the perspectives of patients, physicians, and health care executives for using mHealth systems in Sub-Saharan Africa and to provide recommendations on mHealth system design and policy-making. The study’s anticipated outcome is a description of the elements that encourage or inhibit the use of mHealth approaches, along with suggestions for resolving these barriers and exploring the enablers. This study can be used as a primary step in undertaking a user-centered design study of mHealth platforms in the Sub-Saharan African context.

## Methods

### Study Design

A multi-level and multi-actor in-depth semistructured interview was employed in order to identify the barriers and enablers of the use of mHealth systems.

### Study Area and Sampling

The sample areas for this study included 1 city administration and 2 regions in Ethiopia, namely, Addis Ababa city administration, Oromia region, and Harari region. The study locations were selected based on the Human Development Index (HDI), which serves as a composite measure of a region’s average achievements in 3 fundamental aspects of human development, namely, health, knowledge, and standard of living [28]. Participants were recruited through nonprobability sampling, with a specific emphasis on purposive sampling techniques. For recruiting individuals, the snowball sampling technique [29] was used by means of colleagues, organizational contacts, and initial participants.

### Participants

Data were collected from patients, physicians, and health care executives. For the health care executive group, individuals eligible for participation included decision-making persons and managers of health offices, hospitals, or similar organizations. For the physician group, individuals eligible for participation included health care professionals working at chronic disease outpatient departments (OPDs), who possessed the ability to communicate in Amharic. Lastly, for the patient group, individuals eligible for participation included chronic disease patients aged between 17 and 50 years who could communicate effectively in Amharic. These criteria were carefully defined to ensure that the selection of participants aligned with the specific characteristics and roles of each group within the study.

The participants in the health care executive group had a variety of positions, including office and hospital directors, coordinators, and ICT heads in federal offices, health bureaus, and hospitals. Additionally, in-depth interviews were conducted with physicians working at chronic disease OPDs. Furthermore, patients who visited the selected health facilities for chronic disease follow-ups were involved in the study.

### Data Collection and Analysis

Semistructured interviews [30,31] were selected for this study as they provide the required balance between flexibility and structure for our research. This approach offers the necessary room to explore a subject while maintaining sufficient structure to accomplish the objectives of the study. The interview guides for patients, physicians, and executives are presented in Multimedia Appendix 1, Multimedia Appendix 2, and Multimedia Appendix 3, respectively. The interview guides were structured based on the consolidated framework of the factors impacting clinicians’ adaptation of mHealth [24]. All interviews were conducted in-person with the participants. Prior to conducting the interviews, participants received an information letter explaining the overall goal of the PhD research and this specific study. An oral explanation was also provided where necessary. Informed consent was obtained from each participant in written form, and their participation was voluntary. When confidentiality and privacy could be assured, interviews with patients were conducted in the waiting area of the hospital. Interviews with physicians and executives were conducted at their offices. No monetary compensation was provided to any of the participants. All of the interviews were carried out by a female researcher (author GTA), who is a biomedical engineer and a PhD student with a focus on the design and development of mHealth systems for Sub-Saharan Africa. The researcher has experience and training in various research methodologies, including qualitative study. The interviews were conducted in Amharic and were audio recorded. The audio recordings from the interviews were first transcribed verbatim and then translated to English. No automatic tool was used for transcription. Google Translate was used for translation, and the information was checked manually for correctness. The thematic analysis methodology described by Braun et al [32] was employed for this study. The author GTA coded the interview transcripts and revised them with the 2 researchers JMA and GLS. NVivo software (QSR International) was used during the data analysis to code and categorize the data and to create a thematic framework. To report the study, we used the COREQ (Consolidated Criteria for Reporting Qualitative Research) checklist [33], which has been provided in Multimedia Appendix 4.

### Ethical Considerations

Ethical clearance was obtained from the KU Leuven Social and Societal Ethics Committee (SMEC) (G-2022-5491-R3(MIN)) and from the Jimma University Institute of Health Institutional Review Board (IRB) (JUIH/IRB/311/23).

## Results

### Overview

A total of 48 interviewees participated in the one-to-one interviews. In the patient group, 17 patients (10 men and 7 women) were interviewed. Eight additional patients were approached, but they declined to be interviewed as they were in a hurry, were not willing to be interviewed, or were already frustrated with the system. The mean patient age was 33.9 years, and the mean interview duration was 6.25 minutes.

In the physician group, 19 physicians (12 men and 7 women) were interviewed. Six additional physicians were approached, but they declined to be interviewed as they had hectic schedules. The physicians included in the study were general practitioners who worked in chronic disease OPDs and had an average of 5 years of experience. In this group of individuals, the mean interview duration was 15.7 minutes.

In the health care executive group, 12 health care executives (10 men and 2 women) were interviewed. Owing to a scheduling conflict, 1 additional executive declined to be interviewed. In this group, the mean interview duration was 19.34 minutes. The executives had a variety of positions, including directors in the country’s health minister’s office, directors in city health administrative offices at the zonal and regional levels, hospital chief clinical officers, and ICT heads at hospitals. Owing to the limited number of key respondents in the health care executive group, the number of participants in our study was restricted. Nonetheless, we ensured that the interview process was comprehensive enough to address all crucial aspects, and we included all key informants while also exploring any new viewpoints that emerged.

The total sample size was established based on data saturation, which occurs when new data no longer provide additional perspectives or insights after a certain point. In this study, data saturation was reached following 48 interviews (17 patients, 19 physicians, and 12 health care executives), indicating that an adequate sample size had been achieved. Table 1 provides an overview of the interview characteristics.

**Table 1:** Overview of the interview characteristics.

Variable		Patient group (n=17)		Physician group (n=19)	Health care executive group (n=12)
**Gender, n (%)**					
	Male	10 (59)	12 (63)		10 (83)
	Female	7 (41)	7 (37)		2 (17)
**Age (years), n (%)**					
	20-25	5 (29)	N/A^a^		N/A
	26-30	4 (24)	N/A		N/A
	31-45	5 (29)	N/A		N/A
	>45	3 (18)	N/A		N/A
**Years in practice, n (%)**					
	<5	N/A	6 (32)		N/A
	5-10	N/A	13 (68)		N/A
**Position, n (%)**					
	Director	N/A	N/A		7 (58)
	ICT^b^ head	N/A	N/A		2 (17)
	Coordinator	N/A	N/A		3 (25)
**Location, n (%)**					
	Addis Ababa	6 (35)	7 (37)		5 (42)
	Oromia	6 (35)	5 (26)		4 (33)
	Harari	5 (30)	7 (37)		3 (25)
Interview duration (min), mean (SD)		6.25 (5.76)		15.70 (6.97)	19.34 (8.15)

^a^N/A: not applicable.

^b^ICT: information and communication technology.

A total of 137 determinants for using mHealth systems in Sub-Saharan Africa were identified from the perspectives of patients, physicians, and health care executives. Of the 137 determinants, 68 were barriers and 69 were enablers. Themes were derived from the data. Further categorization of these aspects in the study resulted in the identification of 3 major categories: organizational and policy, social and personal, and technical and material. These categories were based on the consolidated framework of the factors impacting clinicians’ adaptation of mHealth [24], which takes into account the numerous factors influencing adoption of mHealth systems. The organizational and policy factors pertain to health care organizations’ internal workings, workflow, policies, regulations, patient-related factors, and user engagement. The technical and material factors pertain to system design, system usefulness, IT capabilities, compatibility, data management, user experiences, monetary factors, and ease of use. The social and personal factors include personal characteristics, social and cultural factors, and moderating factors.

### Perspectives of Patients

**Figure 1:**
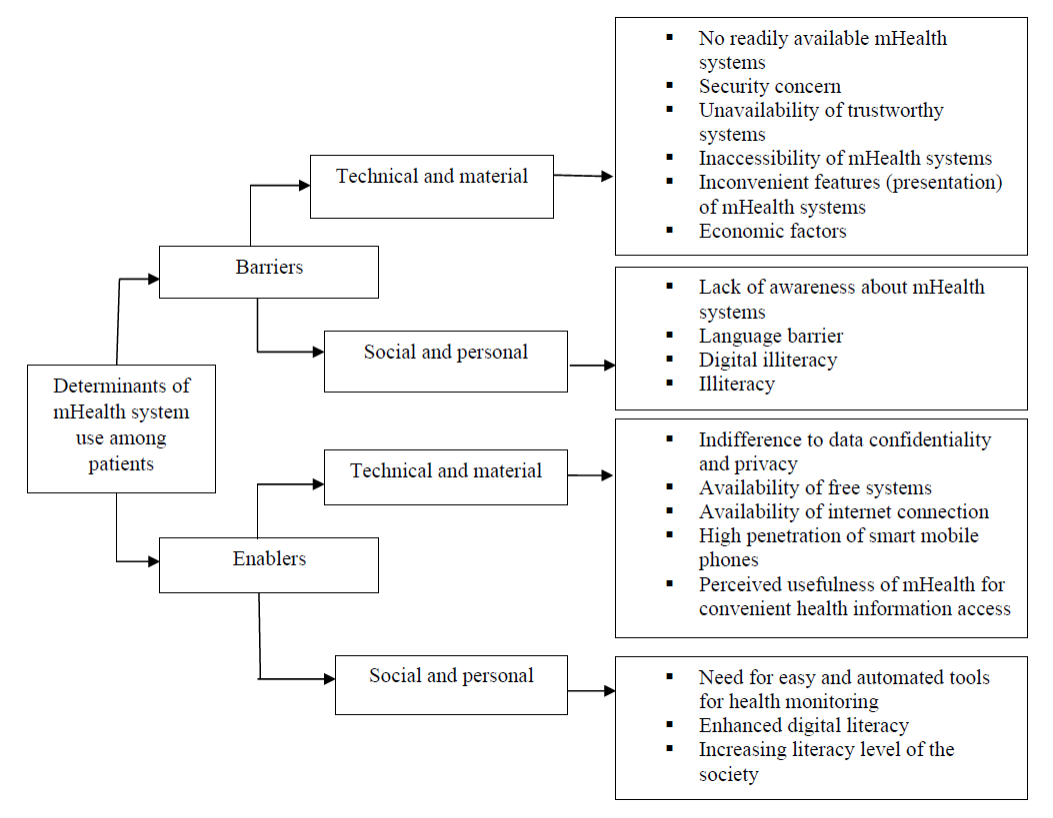
Barriers and enablers for using mobile health (mHealth) systems: patients’ perspectives.

Among patients, after a detailed examination of the data, we identified 10 unique factors that could impede progress, which were referred to as barriers, and 8 unique factors that could facilitate progress, which were referred to as enablers. Figure 1 provides a comprehensive list of these barriers and enablers. Patients were assigned a code based on the study area as follows: those from Addis Ababa were coded “AP,” those from Harari were coded “HP,” and those from Jimma were coded “JP.”

#### Barriers

Among the 10 barriers hindering the adoption of mHealth systems, 6 were under the technical and material category. These challenges included system design, system usefulness, IT capabilities, compatibility, data management, user experiences, monetary factors, and ease of use. The remaining 4 barriers were under the social and personal category, which included personal characteristics, social and cultural factors, and moderating factors.

The majority of patients reported that they have never used mHealth systems. However, most of them reported that they would use mHealth systems if they had awareness and if they could access these systems in an affordable way.

More than half of the participants had no awareness about mHealth systems and how they could benefit them. However, upon a detailed explanation about mHealth systems and their importance, they reported that they recognize the usefulness of such tools. They reported that they would use these systems if they could get trustable systems and if recommended by their doctors.

Regarding the barriers associated with using mHealth approaches, the most frequently cited theme was a lack of awareness about mHealth systems.

I don't have the information about such things either. Apart from seeing the texts that come to me, I never installed the application and tried nothing.

AP4

To be honest... I have never heard or seen health practitioners recommending this to me. I have no idea about such systems.

AP6

I don't know much about that, but I think it is useful for monitoring my diet and sugar levels.

HP3

The knowledge gap… For example, when I see my friends using such things, I don't know the source of it. And I learn from them, but for example, when I am talking about this idea with my friends with whom I have the same interest like me they ask me from where I get it from.

JP2

I don't have the information about this.

JP6

Patients also perceived the fact that mHealth systems are not readily available as a barrier to not using them. They reported that they browse Google or YouTube when they need to obtain information related to health.

I will download some stuff on YouTube. Video… I see…. I am downloading some video.

JP4

The only thing I've used so far is Walk Exercise. It is by just browsing.

AP2

The affordability of smart mobile phones, the requirement for a paid subscription, and the high cost of smart systems were identified as economic factors that can impede the adoption of mHealth systems. These factors are believed to create obstacles for individuals who may not have access to the necessary technology or cannot afford the associated costs.

Because this information is from the internet. It is not possible to access the internet if it is not purchased. Again, you can find that if you go to a place with internet. Even if I have a cell phone, it costs money.

HP1

But the affordability... I can't afford that mobile so I won't use it.

HP1

But with the systems, the smart ones are a little expensive, so the cost of the sensors is a barrier.

AP6

A lack of trustworthy mHealth systems is a significant obstacle to their adoption. Patients feared that the information and services provided by these systems may not be based on reliable evidence. This concern arose from the potential harm caused by inaccurate information or ineffective treatments.

I want it to be something secure. First is the issue of medication. Medical information is not something you can just throw away. Therefore, they have a question of credibility.

AP4

The reason I don't use it is because most of them are not desirable. Because I hear things that are not trustworthy; I won't take advantage of it.

AP5

The application must be supported by evidence.

JP4

Inconvenient features (presentation) of mHealth systems were also perceived as an obstacle to the use of these systems.

I think that a lot of data frustrates people. When you read more data, it is like an education. It needs your time. It needs your opinion. Anyone who is not in the health field may not have an interest in such things.

JP2

The presentation is a biggest barrier... even if it is translated... there are some words that are cultural and from our community language... there is, isn't it... I think there is a small barrier. Medical terms... because it's a bit difficult to how interpret them.

AP6

Digital illiteracy, which refers to the lack of ability to effectively use digital technologies, such as computers, smartphones, and the internet, is a recognized barrier to the adoption of mHealth technologies. This challenge is believed to limit the ability to take advantage of the benefits provided by mHealth tools.

Lack of skill in using mobile phones. Configuring systems specially in IOS it is also same for android is difficult. Like Entering Personal Details. And because the app won't start without you doing it.

AP6

I don't know how to use this kind of thing. I don’t have the knowledge of how to use such systems. So, I never used it.

HP4

#### Enablers

Among the 8 enablers facilitating the adoption of mHealth systems, 5 were under the technical and material category and the remaining 3 were under the social and personal category.

Patients with chronic diseases had a need for easy and automated tools for health monitoring.

It's a matter of health. To control my blood sugar level, there are mandatory things that I have to do. So if there are things installed on my phone to help me with this, it helps.

AP2

I am suffering from hemorrhoids. Some say wash with cold water; Some say to wash with warm water; not to be confused it will be good if there is a tool to use in my phone.

AP3

One of the things that inspired me to use it was to find out how many calories I burned in a day; It reminds me how much I should move, if I don't move, I will be exposed to other related diseases. It means it has health benefit for myself.

AP6

It will be very good. I may not always in need to go to the hospital. I am able to adjust myself from that information; Use the medicine on time. Adjusting the food system; doing activities; There are also recommendations. I have arrived to that conclusion. And it's good. And it's important to have.

HP1

I think it would be helpful to have more of information, especially to monitor my health.

HP4

I think it makes things easier for us. If I use it, it will be beneficial. The usefulness is very high.

JP1

Patients recognized the availability of the internet as one of the facilitating factors of the use of mHealth systems as it could help them access such platforms.

At home, I have Wi-Fi, so it's easy.

AP2

Now there is telephone, there is Internet, there is Wi-Fi, now there is internet even in a pool house, there is even in a tea house.

JP2

There is a big difference between where there is internet and where there is no internet. Internet is a way to get many new technologies. We have it now more than ever.

JP4

Another enabler was the perceived usefulness of mHealth as a convenient tool to access health information.

…. And it helps you to be proactive about your health. It means it prevents you from going to the hospital after something happens.

AP6

### Perspectives of Physicians

Among physicians, we identified a total of 54 factors that were perceived to influence the adoption of mHealth systems. Of the 54 factors, 27 were identified as barriers that hinder the use of mHealth systems and 27 were identified as enablers that facilitate their adoption. The study further classified these factors into 3 main categories, namely, organizational and policy, social and personal, and technical and material. Physicians were assigned a code based on the study area as follows: those from Addis Ababa were coded “AD,” those from Harari were coded “HD,” and those from Jimma were coded “JD.”

#### Barriers

**Figure 2:**
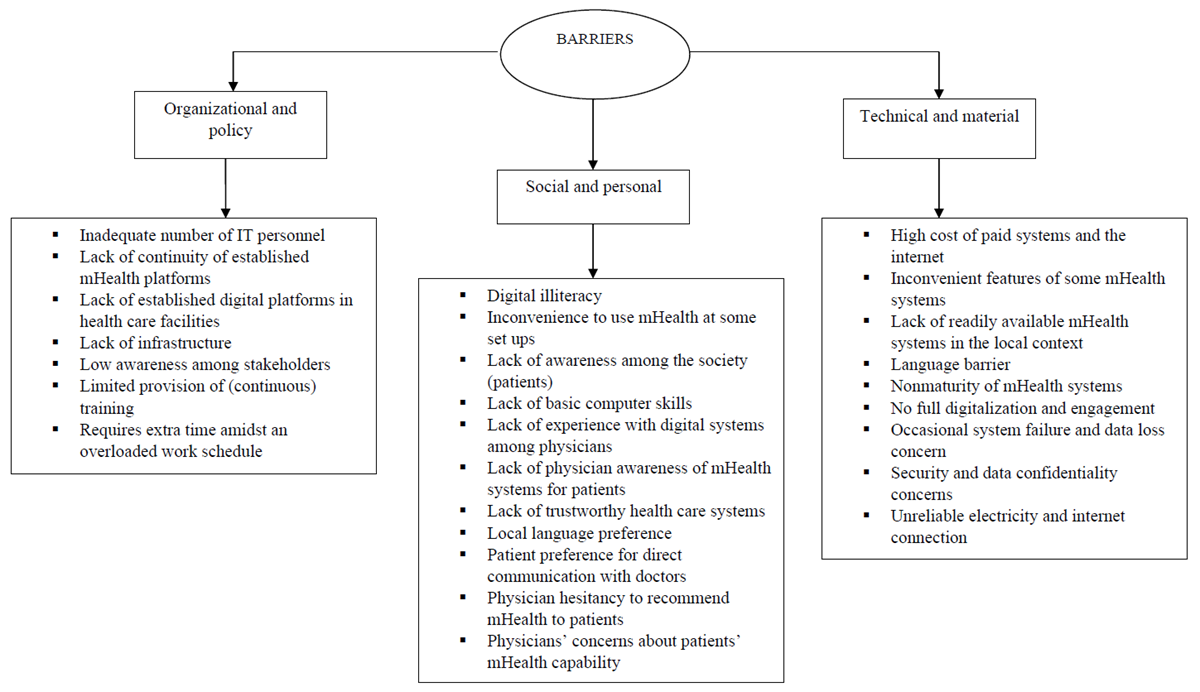
Barriers for using mobile health (mHealth) systems: physicians’ perspectives.

Specifically, among the 27 identified barriers, 7 were under the organizational and policy category, 11 were under the social and personal category, and 9 were under the technical and material category. Figure 2 displays the obstacles to the use of mHealth systems from the viewpoint of physicians.

Physicians identified the lack of established mHealth systems as the primary reason for not using them. They acknowledged that their lack of exposure to these systems has prevented them from gaining experience in their use.

Actually, there is no such system or application based health care at the organization where I work.

HD3

For example, a patient comes to us and we ask about the patient's problem, then we ask and investigate to do a case diagnosis, and at the end we write an investigation paper to investigate and send it to the patient. And we give them hard paper. And we have not prepared them in a computerized way. There is none.

HD6

The main thing is that this type of system does not exist here.

JD2

According to physicians, certain types of mHealth systems have been implemented in some organizations they work for. However, these systems are often interrupted and lack continuity. Physicians considered the discontinuity of established mHealth systems as one of the key barriers to the effective use of mHealth tools.

Around two years ago, this program was being popularized, but nobody in the middle was using it. It is not waited until we become experts. It was interrupted in the middle.

JD5

Sometimes, due to the data and various factors, the system encounters some errors. So we will go back to the manual paperwork at that time. Therefore, when we report using a digital system, if there is an interruption, we often go back to paperwork.

HD7

Another barrier preventing the use of mHealth systems was the required extra time amidst an overloaded work schedule, especially for recommending such systems to patients.

And again, just because there is high patient load here; I don't have that kind of time. It does not allow to say use this or not. As one patient leaves, another enters. There is a load, and it doesn't allow us to have much contact with the patient. We are going to send by diagnosing and treating with what we already have.

AD1

A further barrier to the adoption of mHealth systems was the lack of physicians’ awareness about mHealth systems for patients. Some physicians reported that they are not well-informed about these systems, and even for their own personal health and wellness, they rely on browsing the internet rather than using stand-alone health systems. This lack of knowledge and personal use can limit their ability to confidently recommend and implement mHealth tools in their practice, which in turn can hinder patient adoption and engagement. To overcome this barrier, there is a need for greater education and training programs for physicians on the benefits and effective use of mHealth systems, as well as efforts to increase physician engagement with these tools to help build their confidence and understanding.

Now, for example, I don't know anything personally, but for example, I don't know an app for diet. Honestly, now, in terms of exercise, I use YouTube downloads even for myself.

AD2

Some physicians claimed that they have never given these systems any consideration.

From my point of view, there were no situations where we would recommend using this to my patients. Maybe, I have never thought about it.

JD4

I don't have the knowledge. I mean, I didn't know there was anything like that about mHealth that you just told me about. To tell you the truth, I only found out about Mobile Health Solution today.

AD5

Another barrier was physicians’ concerns about patients’ mHealth capability. They acknowledged being worried that their patients do not have the necessary skills or access to technology to make the most of mHealth tools.

But as I said, it's not that the applications have problems; As I told you before, I don't think anyone will use it. It just doesn't feel like that to me.

AD1

Most of them are illiterate. It is uneducated and I don't think they will use this app.

AD5

As I mentioned earlier, the problem is that the demographics of the patients are too old to use such systems. They may have a little trouble.

AD6

What I would consider being the biggest obstacle... Most of the chronic follow-ups have a low level of education. So they may find it very difficult to use applications.

AD7

I think they are less literate and less qualified. Because the majority cannot read a book. So I think that will be like a gap.

JD2

Physicians mentioned a further obstacle in the adoption of mHealth systems, namely, lack of trustworthy systems, which raises concerns about the potential risks of recommending them to patients.

People with chronic conditions are very bitter, so they search for something to escape. Sometimes there is information that takes you to stop taking medicine. So, this is the side effect of modern digital. It's not even called a limitation.

JD4

Another barrier was the lack of readily available mHealth systems in the local context.

But it is not prepared at the app level in our country. It is better if we use this application prepared like this. We tell patients that they will find it on Google because it is not set up like that.

AD7

The first and most important thing that we can't do is we see it from our country's perspective, if we see it like Ethiopia, there are no rich digital applications for that. Not being able to provide us with that information is the number one factor.

HD7

Most of the time, because those data are based on foreign populations, those findings have nothing to do with the population of our country.

HD1

Security and data confidentiality concerns were also perceived as barriers to the use of mHealth systems.

I usually log in with my Google account. I have already accepted my Google account. And I have doubts. I am not sure. As I don't have details about the system. I don't know much about server security. Some app says you can accept or not about personal issue when you open an app. It may be exposed. I'm not sure about the data security and confidentiality.

HD4

Another barrier was the inconvenient features of some mHealth systems. Most mHealth systems that physicians can use do not have an offline option, and many of these systems work online.

The biggest problem is that they are all online. They don't have offline. And you absolutely need data.

HD4

A lack of reliable internet and electricity was an additional obstacle.

After all, if there is no internet, we cannot get this information. The main obstacle is the internet. Most mHealth applications are internet-based, so information cannot be accessed when the internet is down.

HD7

#### Enablers

**Figure 3:**
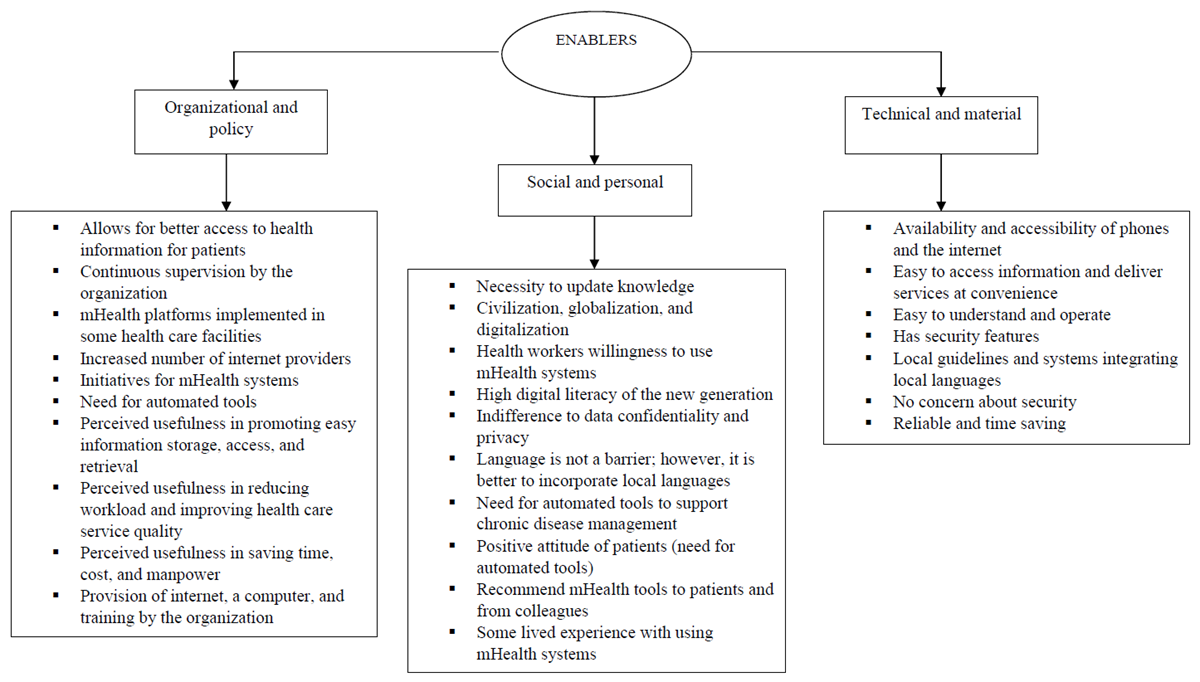
Enablers for using mobile health (mHealth) systems: physicians’ perspectives.

Among the 27 enablers that facilitate the adoption of mHealth systems, 14 were under the organizational policy category. These factors pertain to health care organizations’ internal workings, workflow, policies, regulations, patient-related factors, and user engagement. Moreover, 4 enablers were under the personal and social category involving personal characteristics, cultural factors, and moderating factors. Furthermore, 16 enablers were under the technical and material category encompassing mHealth system design, IT capability, compatibility, user experience, data-related factors, ease of use, and monetary aspects. Physicians’ perspectives on factors that facilitate the use of mHealth systems are depicted in Figure 3.

Physicians perceived usefulness in promoting easy information storage, access, and retrieval as one of the enablers of the use of mHealth systems. Despite limited exposure to these systems, physicians believed that they represent a convenient way to manage patient data, with the advantages of avoiding loss of information and enabling reliable information retrieval.

On the positive side, there is no such thing as loss of information such as manual cards and paper, papers can be torn and lost. Patients can also be abused. But if we use mHealth systems such as EMR, it will not disappear because we will simply be here and send all the investigations. Whether the data is from this year or before, I can check many things about the patient. It makes it easier for me because I have everything I need there.

AD1

You will not lose the file, it is well recorded. What did they have before? Or what medication were they taking? Even if the patients don't know what medicine they are taking, how much dose they are prescribed, how many times a day they are taking, so I can get a record from this.

AD2

Therefore, getting reliable information and then delivering that information to the concerned patient is of great importance. Using mHealth in general allows us to get better information and updated information.

HD7

You will find information in applications in a very easy and understandable manner. There are books in hard copy. Sometimes when you read those hard copies, you don't understand them, when you go with those applications, they are very encouraging because they are easy to understand.

HD3

Knowledge is not a big deal as it used to be. Knowledge is cheap. Knowledge is what you can find anywhere by Googling. It is a matter of reading and not reading. But everything is up in the air. And it's not like you used to go to the library to look for a book and get a book. Everything is on your phone. You can open and use it even during operation. Whenever you want. It's very easy.

HD4

Another enabler was the perceived usefulness in reducing workload and improving health care quality. According to physicians, using mHealth systems has the potential to lessen the strain of health care personnel while also raising the standard of care.

The first reason for not providing quality service is the workload and if something reduces that load, if there are supportive things like this, I think the quality will improve.

AD1

The timing itself. If you have a current patient, what time did she take her medicine? Did she take it or not? She will be evaluated strictly on time. She takes her medicine on time. A professional can't lie if he skips it knowingly or unknowingly. He cannot say that he gave it to her. When you record what you just did, it will record the time.

AD4

You can treat your patient in an international standard way.

HD4

Physicians mentioned that mHealth systems allow for better access to health information for patients. Some believed that providing patients with access to various health-related information at their fingertips will empower them.

Using mHealth in general allows to get better information and updated information. Especially when it comes to health. It helps to live a healthy life by getting new information every day.

HD7

If the patients have it on their hands, I think it will be a daily experience for them too. It will be easily accessible and it will save our time for the patients as well.

HD2

Our patients, who have been treated, do not come back with any complications. Because they can get health information and monitor theirs case. Because they may be able to get every detail on the system.

HD6

Another enabler was the need for a chronic disease management support tool. Physicians recognized the potential benefits of digital automated tools for patients having chronic diseases, as consultations and visits to their office alone may not suffice. To promote healthy lifestyle changes and provide necessary support, it is imperative to equip patients with user-friendly tools that can be integrated into their daily routine.

First, when there is a patient like this... there are chronic ones, for example, diabetic, hypertensive, heart failure. Because they need not only medication but also life modification, they may not achieve what we have told them in one day. But everything how to modify their lifestyle detail is there, so when they get access, they can easily remember what we told them and continue their life. Beyond medication, by the way, also cares about their lifestyle.

HD6

Availability of phones and the internet was also perceived as a facilitator. According to physicians, availability of phones and the internet has tremendously facilitated the adoption of mHealth systems in a variety of ways. First, regardless of location or the time of day, users may readily access health information and services via their mobile phones. Second, health professionals can use digital tools to gather, preserve, and share patient data, which can improve the quality and efficiency of health care services. The increasing use of mobile phones and the internet in Sub-Saharan Africa has created an opportunity to use these platforms to deliver health information and services to individuals who may have limited access to traditional health care services.

This is because there is internet access and broadband, so everyone works connected to the network. Without that, this simple system wouldn't exist.

AD1

Now, for medical and other purposes, because of this technology, because of the advent of smartphones, the spread of Wi-Fi, the advent of the Internet, I think that it has made everything easier even the medical education.

HD4

Having a mobile phone and the internet makes things easier.

HD5

Physicians who have experienced mHealth systems recognized that these systems are easy to understand and operate. They declared that for users who are familiar with technology, these systems are easy to use.

So you need to know how to access the systems. They are mostly easy. There is nothing difficult unless you are someone who is not familiar with technology to use it. You can search. You can log in and access what you want. I don't have anyone who says it's a challenge.

AD6

### Perspectives of Health Care Executives

Among health care executives, we identified 65 factors that were perceived to influence the adoption of mHealth systems. Of these 65 factors, 31 were classified as barriers and the remaining 34 were classified as enablers. Further categorization of these aspects in the study resulted in the identification of 3 major categories: organizational and policy, social and personal, and technical and material. Health care executives were assigned a code based on the study area as follows: those from Addis Ababa were coded “AA,” those from Harari were coded “HA,” and those from Jimma were coded “JA.”

#### Barriers

**Figure 4:**
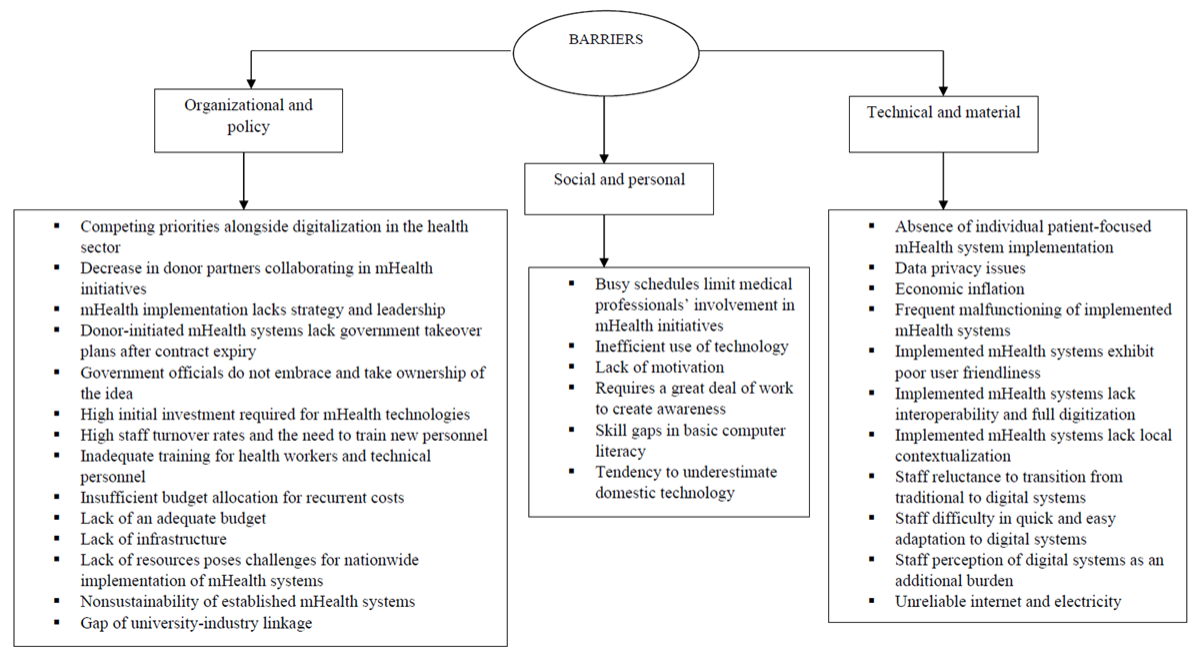
Barriers for using mobile health (mHealth) systems: health care executives’ perspectives.

Among the 31 barriers, 14 were under the organizational and policy category involving elements like the internal working environment of health care organizations, workflow-related challenges, policies and regulations, patient-related factors, and user engagement issues. Moreover, 6 barriers were under the social and personal category involving aspects like social and cultural norms, personal traits, and moderating factors. Furthermore, 11 barriers were under the technical and material category involving difficulties with usefulness, IT capability, compatibility, data-related factors, user experiences, financial factors, and ease of use of mHealth systems. Health care executives’ viewpoints on obstacles to the use of mHealth systems are presented in Figure 4.

From the perspectives of executives, one of the challenges of implementing mHealth systems in Sub-Saharan Africa is the availability of competing priorities alongside digitalization in the health sector. There are competing demands for resources and attention, such as investing in health care infrastructure, improving health care workforce capacity, and addressing immediate health needs.

We are facing multiple health challenges, including infectious diseases as well as non-communicable diseases like diabetes and hypertension. Additionally, the health sector faces critical issues such as inadequate infrastructure, a shortage of skilled healthcare workers, and limited financial resources. Because there are competing priority areas in the sector that require money. From all of them, it is assigned to this sector and these are taken as a challenge not to work completely with hands.

AA5

High initial investment required for mHealth technologies was also perceived as a barrier by executives. The cost of implementing mHealth systems includes not only the purchase of hardware and software, but also the cost of training health care workers, adapting existing health care workflows, and establishing new data management and privacy protocols.

As a problem in supply, mHealth technology is expensive. Not being able to avail of all of them is an issue.

HA3

These start-ups require financing. Although the return is high, the initial investment is also high. mHealth activities require so much investment.

JA2

Another barrier was that some donor-initiated mHealth systems previously implemented in health care facilities lack government takeover plans after contract expiry. These mHealth systems involving donor support do not have clear take over and exit strategies for ensuring sustainability of the projects.

A project has a lifecycle. It comes up with a project by donors and be started that way.. It won't be owned when it is done. It will not be owned by the government.

AA34

Skill gap in basic computer literacy was also perceived as a barrier. These skill gaps can be partly attributed to limited exposure or access to digital devices such as computers.

Computer access is limited. The smaller the access, the less likely it is to use mHealth activities. So there is an obvious skill gap. So does the professional.

JA2

Another barrier was inefficient use of technology.

Efficiently manipulating those technologies if we had them; Proper use of data; One of the challenges is not using technologies properly. We should use them properly and take the appropriate value from them.

AA5

The absence of individual patient–focused mHealth system implementation was perceived as another barrier to the adoption and implementation of mHealth systems in health care.

While some health care facilities have implemented mHealth initiatives, these initiatives are more focused on the institution rather than the individual patient.

The one that we are using on ART is not to be used by the individual patients. It is an application more with professionals on the periphery than with patients.

JA2

A further barrier was that implemented mHealth systems lack interoperability and full digitalization. Although attempts to implement mHealth are sparse in health care facilities, lessons can be borrowed from implementation challenges in other areas, such as electronic medical records (EMRs). Some facilities have deployed EMR systems. EMR systems are not fully digitized, with most of them involving a combination of manual and digital systems. Some health care executives also reported that due to mismatch or lack of integration between some medical devices, they are unable to deploy fully digital systems.

There is a lot of fragmented stuff.

AA34

It is half and half... It is only for reporting, but to make our work more active, it is very good if the facility is fully digital and the delivery points pass through the system through the network.

HA1

Interoperability is not yet implemented. The two systems are not interoperable. The interoperability is being tested for the country.

JA1

We have implemented about 80% of the Laboratory Information System (LIS) in our hospital.

JA2

Another barrier was the difficulty of staff to quickly and easily adapt to mHealth systems.

First of all, sometimes when new things come, there are problems of getting used to that digital thing quickly and not keeping up with it.

JA1

Another barrier was the lack of awareness among staff members. Going fully digital requires a great deal of work to create awareness, as mHealth technologies are relatively new in the region and many health care workers may not be familiar with them.

As a challenge, we still need a lot of attention for technology in general by the government. We are still in the early stages of our own user experience.

AA2

There is an opportunity created by technology. However, creating awareness of the work at all levels, from the leadership to health institutions or to the community should be done. Therefore, one of the biggest tasks is to make the awareness. From top to bottom, the importance needs to be well inculcated. This is the lack of awareness of the decision makers from the community level to the top level.

AA5

The above issue is further exacerbated by the fact that the barrier is related with staff’s reluctance to transition from traditional to digital systems. This lack of awareness and knowledge can lead to resistance or hesitancy in adopting mHealth systems, even if they have the potential to improve patient outcomes and operational efficiency. Health care staff may be unsure of how to use mHealth tools and systems, or they may not understand the benefits of using them.

People who are used to paper work often don't want to be told that we are going to do digital work.

JA2

The biggest thing I have seen is that there is a problem of commitment. It means that it is very challenging for a person to leave what he used to and come to something new.

JA3

#### Enablers

**Figure 5:**
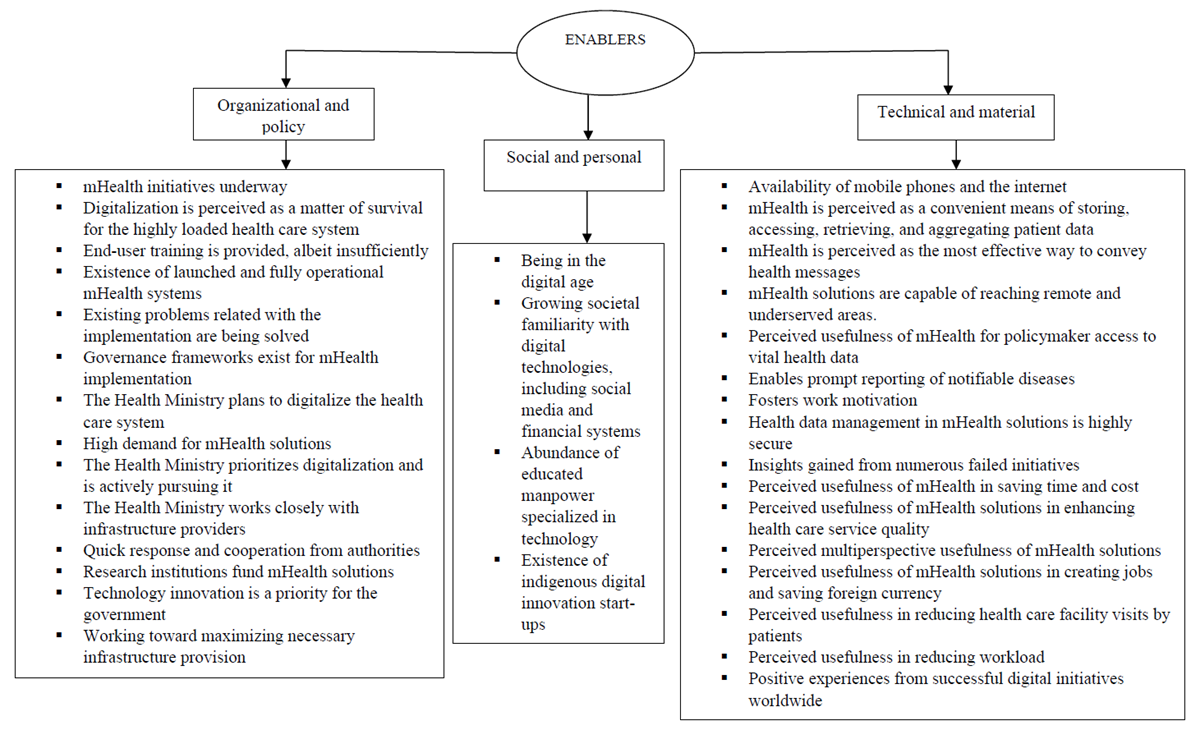
Enablers for using mobile health (mHealth) systems: health care executives’ perspectives.

Among the 34 factors that facilitate the adoption of mHealth systems, 14 were under the organizational and policy category. These factors pertain to health care organizations’ internal workings, workflow, policies, regulations, patient-related factors, and user engagement. Moreover, 4 enablers were under the personal and social category involving personal characteristics, cultural factors, and moderating factors. Additionally, 16 enablers were under the technical and material category encompassing mHealth system design, IT capability, compatibility, user experiences, data-related factors, ease of use, and monetary aspects. Health care executives’ viewpoints on enablers of the use of mHealth systems are presented in Figure 5.

From the perspectives of executives, the health ministry’s plan to digitalize the health care system is one of the main facilitators or enablers of the use of mHealth systems.

There is a direction according to the government. To use such technologies in most cases; It is for online use. The ministry itself has a strategic plan. To digitalize the healthcare system. It is a priority.

AA1

The executives emphasized that the plan is not just a matter of execution but also of giving priority and actively pursuing it.

And now technology is making a big contribution to the health sector. And the ministry understands what a great advantage we have; Departments in the Ministry of Health are working with great initiative using mobile technology.

AA5

Another factor that enables the implementation of mHealth systems is the existence of governance frameworks. Such frameworks were nonexistent a few years ago. However, now, in order to regulate and promote the adoption of mHealth systems, the health ministry has developed a framework that is currently being deployed. Furthermore, a dedicated directorate office has been established to oversee and manage the adoption and implementation of mHealth systems.

Therefore, it is mandatory to create favorable conditions. Facilitating conditions on the ground such as governance frameworks are required. We are working on it. Standards guidelines are required. In addition to this, data standards have also been developed. Now, for example, there are disease codes. A standardized health data access policy has been developed. All these guides the mobile health and govern mHealth as a whole.

AA5

Now, for example, during the time of Covid, There were mandatory situations to initiate telehealth teleconsultation, but if you ask how it works, there was nothing. But now, there is this direction at the level of the Ministry of Health. They have a directorate office.

JA2

Another enabler is the recognition that digitalization is crucial for the survival of the heavily burdened health care system in Sub-Saharan Africa. Hospital executives strongly believed that the failure to digitalize the entire health care system within the facility will ultimately lead to its downfall.

But now, considering the complexity of the treatment and the number of patients we see, the staff is now demanding digital solutions. It is understood that we cannot survive if we do not digitize. We have to digitize the entire operation of the hospital. As I said before, it is a matter of survival. It is a hospital where three thousand four thousand people come and go every day. We have 250,000 to 300,000 outpatients per year. We treat about 20000 people. About 170,000 to 180,000 people will come for emergency. We perform surgery on 15000 people. So this process it is huge process. If this process is not digitized, we cannot provide a smooth service. Because it is causing problems for the patient. As a hospital, we have an assessment that it is increasing the number of deaths.

JA2

According to the executives, another enabler is efforts made toward maximizing the necessary infrastructure provision required for the implementation of mHealth systems. Executives at all levels within the organization realized the importance of providing the required infrastructure, such as ICT and hardware or software resources, to support the deployment of mHealth systems.

In terms of infrastructure... the main thing for digitalization is the internet. They have been given internet access in all nine districts. In terms of computers, they have computers. All adequate internet access to the DHIS health information system, capable computers, and PCs are available at all health centers in the district.

HA2

For example, they can be computers. Servers. It is not directly from the office but it is fulfilled by different partners as they work together with us.

HA3

Along with training, tablets are distributed along with resources such as tablets in areas where they are needed.

JA1

We have invested a lot in infrastructure. Especially our hospital as a health institute has a large ICT infrastructure. We deployed computers. We are working on servers and databases. We trained people and experts for that purpose... It is itself strategically supported and supported by infrastructure.

JA2

Another enabler is the growing societal familiarity with digital technologies, including social media and financial systems. As more individuals become comfortable with using digital technologies in their daily lives, they may also be more open to using mHealth systems.

At the same time, the opportunity is also very profitable because the number of users of social media is increasing from time to time.

HA3

Mobile is now used by many people for money transfer. A lot of things... even if a mHealth system doesn't expand, in the finances... a lot... you sit at home and pay. You can withdraw without an ATM machine. It's simple, a person in finance has experience elsewhere. Mobile is now used by many people for money transfers. It is one exposure to general digital systems.

JA2

The abundance of educated manpower specialized in technology was also perceived as an enabling factor for the adoption of mHealth systems in Sub-Saharan Africa. With a growing pool of technology professionals in the region, there is a greater capacity to develop and implement mHealth solutions that are tailored to the specific needs of the region. This includes the development of innovative technologies, such as mHealth systems and telemedicine platforms, which can improve access to health care services and support patient care. In addition, the presence of a skilled technology workforce can support the deployment, maintenance, and troubleshooting of mHealth systems, which can improve the overall reliability and sustainability of these systems.

… there are many educated young people who can do a lot of creative work; Universities have expanded significantly.

AA2

… Because educational institutions at the national level can easily produce graduates who can easily design and develop these emerging technology applications.

AA5

We also have an ICT director with us. We have teams working on software development. So, as a university, as a health institute, as a medical center, we are doing this work. Second, we are building human capacity. Many students are studying. Despite the quality, I think we have that capacity human power.

JA2

Executives perceived that mHealth solutions are capable of reaching remote and underserved areas where traditional health care services are not readily available, which is a significant enabler for the adoption of mHealth systems in Sub-Saharan Africa. Furthermore, mHealth solutions were thought to aid in overcoming the scarcity of health care personnel by providing remote consultation, training, and education to health care workers, as well as assisting in the delivery of health care services in places with limited resources.

In terms of accessibility, the technology is generally accessible to all communities. The community of our country is located in a very remote place and access to health facilities is very limited. Therefore, mobile technology can be used to make these sections of society that are accessible at different distances easily accessible. Secondly, as mentioned, we have a shortage of health professionals. It is very important to make the service available remotely even if our experts are very limited. From this perspective, it is very important for a society like Ethiopia where there is a shortage of health professionals and the most accessible health facilities are few.

AA5

Executives perceived mHealth solutions as useful tools for enhancing health care service quality, which is a significant enabler for the adoption of mHealth systems in the region. Health care providers can improve the quality and efficiency of health care services by embracing mHealth technologies, such as electronic health records, clinical decision support systems, and telemedicine platforms. Real-time data collection, monitoring, and analysis enabled by mHealth technologies can also influence evidence-based decision-making and assist health care providers in providing fast and accurate diagnoses and treatment plans.

For example, the patient can take advice without coming to the facility. Alerts can go be sent to him. You will find a lot of information. It means that they will not be abused by looking for their doctors. And it generally makes things easier.

AA1

It's easy. It's very simple. It makes it easier. It will be easy for everything in time and for the number of people. So it is very good if we use and serve. It is from two sides. Client side and provider side. It also reduces other errors. Another thing is that it reduces time. So it's very good.

HA1

It will help us gain efficiency. As mentioned earlier, efficiency gains for the health care system are linked to access, mortality, and quality.

JA2

Another enabler perceived by executives is the availability of mobile phones and the internet. The increasing availability of mobile phones and the internet in Sub-Saharan Africa has created an opportunity to use these platforms to deliver health information and services to individuals who may have limited access to traditional health care services.

The fact that the majority of the society is using mobile technology gives the ministry a great opportunity to implement the system. Second, mobile accessibility is expanding. In Ethiopia, it is said that quite a large number of people have mobile phones in their hands. Therefore, the service can be accessed without coming to the health facility.

AA5

For example, if you visit the OPD in a hospital, you will find very few people without a smartphone. If you want to buy a laptop and buy a tablet, It is easy. It is available.

JA2

The overlapping barriers and enablers among the various stakeholders are summarized in Tables 2 and 3, respectively.

**Table 2:** Overlapping barriers among the various stakeholders.

Barrier	Reported by patients	Reported by physicians	Reported by health care executives
Lack of readily available or established mHealth^a^ systems	Yes	Yes	No
Security concern	Yes	Yes	Yes
Unavailability of trustworthy mHealth systems	Yes	Yes	No
Inconvenient features (presentation) of mHealth systems	Yes	Yes	No
Lack of awareness about mHealth systems	Yes	Yes	No
Digital illiteracy	Yes	Yes	No
Lack of continuity of established mHealth platforms	No	Yes	Yes
Lack of infrastructure	No	Yes	Yes
Lack or limited provision of training	No	Yes	Yes
Lack of available mHealth systems in a local context	No	Yes	Yes
Unreliable electricity and internet connection	No	Yes	Yes

^a^mHealth: mobile health.

**Table 3:** Overlapping enablers among the various stakeholders.

Enabler	Reported by patients	Reported by physicians	Reported by health care executives
Indifference to data confidentiality and privacy	Yes	Yes	No
Internet and mobile phone availability	Yes	Yes	Yes
Need for easy and automated tools	Yes	Yes	No
Perceived usefulness in promoting easy information storage, access, and retrieval	No	Yes	Yes
Perceived usefulness of mHealth^a^ systems in saving time, cost, and manpower	No	Yes	Yes
Perceived usefulness of mHealth systems in improving health care service quality	No	Yes	Yes

^a^mHealth: mobile health.

## Discussion

### Principal Findings

The barriers and enablers of the implementation of mHealth solutions in Sub-Saharan Africa were identified and explored in detail in this study. All interviewees (patients, physicians, and health care executives) recognized the potential benefits of mHealth. Several overlapping barriers and enablers were identified among the 3 participant groups.

Lack of awareness about mHealth solutions was highlighted as a common barrier to implementing mHealth systems by patients and physicians. They claimed that it was preventing them from experiencing and using mHealth services. They emphasized the importance of raising awareness among stakeholders. Developers and advocates of mHealth solutions must give outreach and education efforts top priority in order to overcome these issues and increase public knowledge of the potential advantages of these solutions. This might entail collaborations with health care institutions, neighborhood-based outreach initiatives, and population-specific marketing plans. Additionally, in order for health care professionals to use mHealth solutions efficiently and explain the advantages to their patients, they need to receive proper training and support. Finally, overcoming cultural barriers will call for sensitivity to the distinctive cultural perspectives and beliefs of various populations, as well as the customization of outreach and education initiatives [11,34].

Another overlapping challenge noted by patients and physicians is digital illiteracy, which refers to a lack of competency or expertise in using digital technologies, such as computers, the internet, and other digital devices or tools. The good news is that an improving literacy level in the society was also viewed as a facilitator by patients and clinicians. According to physicians, the younger generation’s strong digital literacy level is a tremendous opportunity to leverage and apply mHealth systems. The perspective of executives that supports this view is their observation that society is becoming increasingly accustomed to digital technologies, including social media and financial applications. A study involving cancer survivors reported that low digital literacy may hinder information acquisition and technology-enabled cancer care. The study recommended that digital interventions should be adaptable to varying levels of digital health literacy. Policymakers in health care should acknowledge digital disparities and create targeted initiatives to narrow the digital divide while also meeting the pressing demand for the digitization of health care services [25]. Using digital health information resources and engaging in digital interactions with health care providers offer significant advantages, holding the potential to enhance the efficiency, quality, and accessibility of health care systems, all while empowering patients [26,35].

Another hindrance mentioned is the lack of incorporation of local languages and contextual factors, such as demography, culture, and population, in mHealth systems. Physicians reported that while they are willing to use mHealth systems developed in foreign languages, they prefer those available in the local language. In contrast, all patients interviewed expressed a preference for mHealth systems that incorporate one or more local languages. Studies have also reported the positive impact of user-centric design and local contextualization of mHealth approaches for improved uptake [36,37].

The challenge in engaging patients for accessing diverse patient populations for education or engagement remains an issue. The study by Martin [38] described barriers for patient engagement, including literacy, access to hard-wired technologies, and understanding of an increasingly complex network of medical care. Developers of mHealth systems need to concentrate more on patient-centered design, involve users in the development process, and work to deliver a customized user experience in order to overcome these difficulties. This can make mHealth systems more usable, effective, and accessible for patients, which will ultimately improve patient outcomes. Additionally, by educating patients on the use of mHealth systems, offering support for their use, and highlighting the advantages of these systems for patient care, health care organizations and providers can encourage patients to use them.

Aside from not being aware of mHealth systems, those who have been exposed to the technology are concerned about the availability of trustworthy systems. Because health is a sensitive issue, patients mentioned that if doctors recommend it during their follow-up, they could use it. However, the majority of doctors said that they had never recommended such systems to their patients. This is due to 2 major reasons. First, physicians have little expertise with mHealth systems, which limits their capacity to use them. Second, because of a lack of understanding of patient-focused mHealth systems, physicians are often unaware of mHealth systems that they could recommend to their patients. According to recent research conducted in developed countries, the findings indicate that health care professionals do not endorse the use of mHealth systems to their patients [39]. Nonetheless, the aforementioned study revealed that while health care professionals do not recommend mHealth systems to their patients, they do inform them about the existence of these systems should they express a desire to use them. In contrast to the notion that health care professionals solely remind their patients about the availability of mHealth systems, the findings of this study suggest that physicians harbor a degree of skepticism regarding the ability of their patients, particularly those who are elderly or possess limited education, to effectively use such technologies. Consequently, these physicians generally do not recommend mHealth systems to individuals having chronic diseases. The other reason, as perceived by physicians, is that they could not find trustworthy health care systems to prescribe to patients confidently. The lack of adequate regulation and control is one of the key reasons for the unreliability of mHealth systems. Numerous systems make efficacy claims without any supporting data from the scientific community [21,40]. This may cause consumers to rely on incorrect or partial information, which could be harmful to their health.

Even though security issues were mentioned as common barriers by all the stakeholders in the study, some respondents also expressed different beliefs regarding data confidentiality and security. Some of the participants in both patient and physician groups were not worried about security. Some believed that the system they are using has security features, and some mentioned that they are not worried at all whether the system is secured. However, some patients mentioned that they are not sure whether data confidentiality will be maintained during the use of such systems and said that they are skeptical about it. They recommended better security features while designing such systems. The potential for data privacy violations is another problem. Some systems collect users’ private health information but fail to adequately safeguard it from unauthorized access by outside parties. Users may experience serious repercussions as a result, such as discrimination and identity theft. It is crucial to set precise standards and guidelines for mHealth systems in order to address these problems. Researchers have also suggested ways to underpin a radical rethinking of information privacy, confidentiality, security, and integrity to unlock the potential of mHealth and ensure verified access to often sensitive data [41]. Given the rapid pace of technological development, the protection of personal health information stored in mHealth solutions is an important consideration. In order to protect people’s privacy, it is essential to ensure the confidentiality of such data. This is especially important in light of laws like the Global Data Protection Regulation (GDPR) [42,43].

All patients, physicians, and health care executives perceived the widespread availability of the internet, mobile phones, and digital devices as creating a favorable opportunity to integrate mHealth systems. Though the Sub-Saharan African region is way behind other regions in employing mHealth activities, there is now a high level of commitment and a strong strategic plan to benefit from this opportunity. Furthermore, there is a high need for an individualized patient-focused mHealth system for managing chronic conditions such as diabetes and hypertension. A study conducted by Doyle et al [27] concluded that implementing mobile phone–based interventions is viable; however, there is a risk of exacerbating inequities, particularly if these interventions necessitate internet access. Internet-based mHealth approaches should carefully assess potential risks for participants and include skill-building sessions on secure internet and phone usage.

The Sub-Saharan African region’s population is growing at an alarming rate, and the region is facing the double burden of communicable and noncommunicable diseases. As a result of these, access to high-quality health care systems may be jeopardized. To make problems worse, social distancing necessitated by the COVID-19 outbreak made face-to-face consulting and care-taking difficult in many cases. According to health care executives, integrating digitalization in health care systems is becoming increasingly important for the health care sector, which is under high pressure. Sub-Saharan Africa faces numerous health care challenges, including limited access to health care services, inadequate health care infrastructure, and a shortage of health care professionals. The COVID-19 pandemic has also highlighted the need for mHealth solutions that can help provide care remotely [44].

Digitalization can help address these challenges by improving access to health care services, enabling remote consultation and telemedicine, and improving the accuracy and completeness of patient records. This can help reduce the burden on health care facilities and make it easier for patients to access care, especially in remote or underserved areas. Physicians believed that employing a mHealth system can help to reduce workload and also help to achieve quality health care services. Other scholarly works have also affirmed that digital technologies contribute to improved efficiency and the streamlining of health care services. The potential of mHealth to provide health information to patients and the accessibility of patient data through EHRs make the situation easier for health care practitioners, reducing administrative tasks and enhancing care coordination [10,45,46].

According to patients, a lack of easily available mHealth systems is also a hindrance. This viewpoint was also shared by both physicians and executives. Physicians reported some experience in using mHealth systems such as reference guidelines, gestational age calculators, etc. These mHealth systems, however, lack local context. Furthermore, physicians reported that they were unable to find standalone systems in the form of apps that would allow them easy access to vital information to update their knowledge and fill knowledge gaps. Despite the fact that the integration of mHealth in health care facilities is not widespread, it is possible to draw on the experiences and obstacles encountered during the implementation of other technologies, such as EMR systems. EMR systems deployed in some health facilities have a lot of challenges for use to the full extent possible. However, owing to discontinuity and nonmaturity of the systems, they are not fully functional and some of them have stopped working. This is also perceived as another hindering factor. Despite efforts to launch mHealth systems, health care executives indicated that most launched mHealth systems so far are for health care personnel. Governments, health care organizations, and technology firms must collaborate to develop infrastructure, raise money, lower regulatory barriers, and encourage user adoption of mHealth systems in order to address these issues. By doing this, we can guarantee that mHealth systems are made more accessible to individuals around the world and contribute to improving access to health care services. A body of literature indicated that health care providers face challenges in improving digital health applications, and collaborating with stakeholders for value creation remains a significant obstacle. Despite these challenges, involving stakeholders and addressing their needs could promote the sustainable development of digital health services [47].

According to reports from executive stakeholders, many attempts were made to implement mHealth systems in various health care facilities, but most of them were unsuccessful due to a range of issues such as inadequate government ownership, insufficient budget for recurring operational costs, lack of training, insufficient infrastructure, and lack of interoperability. A recent systematic review of systematic reviews indicated that infrastructure, lack of equipment, and technology gaps together accounted for barriers to the use of mHealth systems in developing countries [48]. Despite these challenges, there were several valuable lessons, such as the need for allocating a sufficient operational budget, proper takeover of projects, and engaging well-matured systems through learning from failed projects. As a result, there is now a renewed focus on digitalization in health care, and mHealth solutions are viewed as valuable tools for addressing complex health care challenges from multiple angles.

A multitude of analogous studies have examined the obstacles hindering the adoption of mHealth systems within health care facilities. Many studies reported lack of knowledge of mHealth systems, infrastructure, lack of equipment, and technology gaps as barriers, and identified the ubiquity of smartphones and apps as a facilitator, especially for the younger generation [39,48]. Absence of a national policy on mHealth, poor internet connectivity, and shortage of electricity were also highlighted as important inhibiting factors for mHealth adoption in low and middle income countries [49]. The perceived usefulness of mHealth approaches among patients has been reported [50]. Some of the enablers identified in this study were introduced by previous studies, such as perceived usefulness in improving health service quality and perceived ease of use [51].

One of the common barriers as perceived by all the stakeholders is security concerns. The results of this study are consistent with the findings of other studies reporting that mHealth app users have security and privacy concerns. The study reported that lack of security features in mHealth apps was a barrier for adoption [52,53].

In the literature, knowledge and limited literacy were presented as barriers for mHealth adoption. However, in this study, an increasing literacy level of the society was identified as one of the enablers. This same factor was reported as a barrier in another similar study [53]. Consistent with the results in this study, a prior study reported that limited digital literacy and the unreliability of health information from mHealth platforms are barriers for mHealth use [25].

Developing mHealth solutions that are suited to the local environment, raising awareness, offering adequate training, assigning adequate funding, incorporating various security features, and putting in place and implementing simple governance principles are a few ways to tackle the challenges.

This study is the first of its kind to provide a comprehensive exploration of the barriers and enablers of the use of mHealth systems in Sub-Saharan Africa, with a focus on the multi-level and multi-actor perspectives of patients, physicians, and health care executives. The barriers identified in this study highlight the challenges and limitations that must be overcome in order to successfully implement mHealth systems in Sub-Saharan Africa. Understanding these barriers can help inform the design and implementation of mHealth solutions that are tailored to the specific needs and context of the region. Addressing these challenges necessitates increased investment in mHealth infrastructure, health care worker training programs, and financial sustainability methods for mHealth initiatives. If these barriers are effectively addressed, it may become possible to overcome the challenges associated with implementing mHealth systems in the region, thereby unlocking the full potential of mHealth to enhance health care outcomes for patients, health care providers, and policy makers. The identified enablers will be further investigated and considered in the design of future mHealth platforms for studies in Sub-Saharan Africa.

### Limitations of the Study

This study is subject to certain limitations, notably the relatively modest sample size, which may restrict the extent to which the findings can be extrapolated to the broader population. Nonetheless, efforts were made to address this concern by selectively recruiting participants from diverse age cohorts and employment backgrounds. Other limitations include a small patient population (the disease may have influenced the patients’ perceptions). Future investigations could benefit from expanding the sample size to enhance the representativeness of the outcomes. Another potential limitation of this study is the absence of triangulation of results. However, steps were taken to mitigate this shortcoming by collecting sufficient data to comprehensively comprehend the phenomenon, thereby enhancing the validity and reliability of the findings.

### Conclusion

The use of mHealth systems in Sub-Saharan Africa has been hindered by a range of factors and has also been facilitated by various enabling factors. Patients, physicians, and health care executives identified common barriers and enablers to the uptake of mHealth systems. The identified barriers must be actively mitigated through the involvement of all relevant stakeholders. Despite the existing barriers, the findings of this study provide a promising outlook for the implementation of mHealth systems in Sub-Saharan Africa. The study highlights the numerous opportunities that exist for the successful integration of mHealth systems into the region’s health care systems. To ensure maximum uptake, it is crucial to adopt a user-centered design approach in mHealth system design and development.

The results of this study have important implications for both mHealth system design and policy-making. The identified barriers and enablers can serve as a guide for the design of mHealth systems that are tailored to meet the needs and preferences of patients, physicians, and health care executives. Additionally, the study findings can inform policy makers on the necessary steps to be taken to facilitate the successful integration of mHealth systems into health care systems in Sub-Saharan Africa.

In conclusion, some of the barriers and enablers of the uptake of mHealth systems in Sub-Saharan Africa are interconnected and require the active involvement of all stakeholders to be addressed. The study provides valuable insights that can inform mHealth system design and policy-making, with the aim of facilitating the successful integration of mHealth systems into health care systems in Sub-Saharan Africa.

## Appendix

Multimedia Appendix 1 Interview guide for patients.

Multimedia Appendix 2 Interview guide for physicians.

Multimedia Appendix 3 Interview guide for health care executives.

Multimedia Appendix 4 COREQ (Consolidated Criteria for Reporting Qualitative Research) checklist.

## Abbreviations

EMR: electronic medical record
ICT: information and communication technology
mHealth: mobile health
OPD: outpatient department
UTAUT: Unified Theory of Acceptance and Use of Technology
