# Supplementary material for: Assessment of the Barriers and Enablers of the Use of mHealth Systems in Sub-Saharan Africa According to the Perceptions of Patients, Physicians, and Health Care Executives in Ethiopia: Qualitative Study
Source: J Med Internet Res. 2024 Mar 27;26:e50337. doi: 10.2196/50337 (PMC11007608; doi:10.2196/50337)
Supplement: Multimedia Appendix 1 [file jmir_v26i1e50337_app1.docx]

**Multimedia Appendix 1. Interview guide for patients.**

**Introductory question**

*1. Please tell me what you know about mHealth solution in healthcare technology?*

**Organization and policy**

*2. How do you explain the readiness/attitude of your healthcare practitioner and yourself in using mHealth systems? Has your doctor ever recommended mHealth for you to use?*

**Technical and material**

*3. Please tell me if you ever have used mHealth systems/apps in your device?* ***(if the answer is I don’t use/never did, then skip question number 4,5,6 and 14)***

*4. What kind of mHealth system do you have/had?*

*5. What encouraged you to have the mentioned mHealth system?*

*6. What more thing/feature would encourage you to have the mentioned mHealth system?*

*7. What has kept you from having mHealth systems/apps on your device?*

*8. How confident are you on the data security/confidentiality issue in using mHealth platforms?*

*9. How mobile/Internet penetration affected the use of mHealth systems in your healthcare facility / yourself?*

*10. How do you think the language that such systems operate helps/hinders the use of mHealth platforms?*

*11. How do you explain the way information is communicated/displayed in mHealth systems?*

**Social and personal**

*12. Please tell us if you experience difficulty in using mHealth systems?*

*13. How do you explain the easiness/difficultness of mHealth system?*

*14. How well can you understand the information on the mHealth system you using?*

*15. What kind of skill gap issues do you think should be addressed?*

**Closing question**

*16. Please share us your view of mHealth system implementation barriers?*
