# Supplementary material for: Assessment of the Barriers and Enablers of the Use of mHealth Systems in Sub-Saharan Africa According to the Perceptions of Patients, Physicians, and Health Care Executives in Ethiopia: Qualitative Study
Source: J Med Internet Res. 2024 Mar 27;26:e50337. doi: 10.2196/50337 (PMC11007608; doi:10.2196/50337)
Supplement: Multimedia Appendix 2 [file jmir_v26i1e50337_app2.docx]

**Multimedia Appendix 2. Interview guide for physicians.**

**Introductory questions**

1. Please tell me what you know about mHealth solution in healthcare technology?

**Technical and material**

2. Please tell me if you ever have used mHealth systems/apps in your device? ***(if the answer is I don’t use/never did, then skip question number 3,4,5, and 16)***

3. What kind of mHealth system do you have/had?

4. What encouraged you to have the mentioned mHealth system?

5. What more thing/feature would encourage you to have the mentioned mHealth system?

6. What has kept you from having mHealth systems/apps on your device?

7. How confident are you on the data security/confidentiality issue in using mHealth platforms?

8. How mobile/Internet penetration affected the use of mHealth systems in your healthcare facility / yourself?

9. How do you think the language that such systems operate helps/hinders the use of mHealth platforms?

10. How do you explain the way information is communicated/displayed in mHealth systems?

**Organization and policy**

11. What does your organization do/provide to support your use of mHealth systems?

12. How does the use of mHealth affect/improve the workflow and workload in your everyday activity?

13. How do you explain the readiness/attitude of patients in using mHealth systems? Have you ever discussed about mHealth with you patients?

**Social and personal**

14. Please tell me if you experience difficulty in using mHealth systems?

15. How do you explain the easiness/difficultness of mHealth system?

16. How well can you understand the information on the mHealth system you using?

17. What kind of skill gap issues do you think should be addressed?

**Closing question**

18. Please share me your view of mHealth system implementation barriers and opportunities?
