# Supplementary material for: Assessment of the Barriers and Enablers of the Use of mHealth Systems in Sub-Saharan Africa According to the Perceptions of Patients, Physicians, and Health Care Executives in Ethiopia: Qualitative Study
Source: J Med Internet Res. 2024 Mar 27;26:e50337. doi: 10.2196/50337 (PMC11007608; doi:10.2196/50337)
Supplement: Multimedia Appendix 3 [file jmir_v26i1e50337_app3.docx]

**Multimedia Appendix 3. Interview guide for health care executives.**

**Introductory / General question**

1. Please tell me what you know about mHealth solution in healthcare technology?

2. Have you ever implemented mHealth systems/apps in your organization?

3. What do you think lacks among healthcare workers to adopt/use mHealth system?

**Organizational and policy**

4. What does your organization do to create an environment that can facilitate mHealth adaption and implementation?

5. Are you aware of any policies that advocates the use of mHealth system? If so, can you tell me more about it?

6. What kind of policies is your office following in improving healthcare service access with regard to mHealth?

7. What is planned/ was done to expand the readiness of healthcare workers to adopt the implementation of mHealth systems?

**Technical and material**

8. How is your office readiness to financially support the adoption/implementation of mHealth systems in Ethiopian healthcare organizations?

9. What infrastructure are arranged in the healthcare facilities to adopt/implement mHealth systems?

10. How do you explain the importance of mHealth to health outcome?

**Closing question**

11. Please share me your view of mHealth system implementation barriers and opportunities?
